# Supplementary material for: Evaluation of the engineering properties of asphaltic concrete composite produced from recycled asphalt pavement and polyethylene plastic
Source: PLoS One. 2024 Apr 17;19(4):e0294179. doi: 10.1371/journal.pone.0294179 (PMC11023403; doi:10.1371/journal.pone.0294179)
Supplement: Attachment — Submitted filename: Response to Reviewers.pdf [file pone.0294179.s001.pdf]

REVIEWER #1

Reviewer's query:

1. Add problem statements.

2. Mention which layer of pavement can be applied according to the obtained results, in terms of stability and gradation.

3. Add aggregate gradation according to standard specification.

4. The value of abrasion test is more than 30%, is there any reasons for that explain.

5. What is the main contribution of this work according to the previous related work.

6. Add recommendations.

7. Explain from your point of view, can we use the RCA in wearing layers for pavement.

8. What about long-term pavement age .. is it suitable or not.

9. I don't see the long term aging test, is there any reason for that.

Authors' Rebuttal and (point by point) Response:

1. **Response:** Thank you for your observation We have now added problem statement(s) to the manuscript on page 6, line 86-87.

2. **Response:** Thank you for your suggestion. We have now included the suggested information on page 8, line 186-187.

3. **Response:** Thank you for your observation. We have added gradation scale on page 6, line 136 while a reference to this table has been added in-text on page 5, line 123-124.

4. **Response:** Thank you for your observation. The value of the abrasion test being more than 30% for both natural aggregates and RAP might be due to the age of the recycled asphalt pavement and the quality of the natural aggregates selected as abrasion test is an indication of resistance of the aggregates to abrasion and impact.

5. **Response:** This work builds on previous studies by combining conventional mechanical analysis with micro-structural and crystallographic analysis of asphaltic concrete to assess its suitability as a pavement wearing course.

6. **Response:** Thank you for your suggestion. We have now added recommendation regarding the need for further study on the long-term degradation analysis.

7. **Response:** Yes, I believe that based on the results of this study, asphaltic concrete sample with the optimum mix meet the specified standard for use in flexible pavement wearing course.

8. **Rebuttal:** Thank you for your suggestion. However, as the long-term degradation analysis was beyond the scope of our work, we have therefore decided to add this to our recommendation for future work in the conclusions section on page 17, line 329.

9. **Rebuttal:** Thank you for your suggestion. As we have earlier stated, the long-term or ageing analysis was outside the scope of our study. Therefore, we have decided to include it as a recommendation for future work.

REVIEWER #2

Reviewer's query:

1. Correct the line no 74 on page no 3 "Similar communications by Ezemenike et al. and Olukanni et. al (6,7) reported tfindings on the" to "Similar communications by Ezemenike et al. and Olukanni et. al (6,7) reported findings on the".

2. In the lines 118 to 128 on the page no 5, addition of HDPE and LDPE is described. However, it is recommended to describe the mixing techniques with conditions.

3. The aims and objectives of the research articles are limited to components of Marshal stabilty of asphalt mixes and microscopic analysis through SEM and XRD techniques. It would be more reliable to perform few asphalt mixture performance test to investigate the performance life with addition of modifiers. It is recommended to add at least one performance test.

4. Physical properties of bitumen are described in Table 1 on page 5. However, the physical testing after addition of HDPE and LDPE are not added. It is recommended to add such results for indication of modification.

5. The conclusion section requires more deliberated results on the with the scope of study.

6. There is no technique applied for the analysis of results. It is recommended to add statistical analysis for better application of results.

7. There are only few references in the introduction section. It is recommended to add more reference and data relevant to scope of the study.

Authors' Rebuttal and (point by point) Response:

1. **Response:** Thank you for your observation We have corrected the typo noticed in our revised manuscript.

2. **Response:** Thank you for your observation. We have revised the manuscript to include the asphaltic concrete preparation method on page 5, line 126-129.

3. **Rebuttal:** Thank you for your observation and suggestion. However, we strongly feel that the chosen test to assess the suitability of the asphaltic concrete recommended by the regulatory body for road construction in Nigeria is sufficient. We also believe that the insights gained from SEM and XRD analysis provide a better understanding of the structure of the asphaltic concrete and its influence on the mechanical properties. We are however open to including additional performance tests in our future work.

4. **Rebuttal:** Thank you for your observation and comments. We feel that the characterization is only relevant to the materials employed for the study. The asphaltic concrete is better characterized in terms of the performance requirements for the anticipated use (Marshall stability, X-ray diffraction, SEM etc.).

5. **Response:** Thank you for your suggestion. We have now revised the conclusions section to include more results that fit within the scope of the study.

6. **Rebuttal:** Thank you for your observation. However, we feel that presenting the results in simple tabular form and statistical graphs is sufficient for our readers to understand our main findings.

7. **Response:** Thank you for your observation. We have now added more citations to the reference section.
